# Design and Implementation of High-Performance ECC Processor with Unified Point Addition on Twisted Edwards Curve

**DOI:** 10.3390/s20185148

**Published:** 2020-09-10

**Authors:** Md. Mainul Islam, Md. Selim Hossain, Moh. Khalid Hasan, Md. Shahjalal, Yeong Min Jang

**Affiliations:** 1 Department of Electronics Engineering, Kookmin University, Seoul 02707, Korea; mainul.islam@ieee.org (M.M.I.); khalidrahman45@ieee.org (M.K.H.); mdshahjalal26@ieee.org (M.S.); 2 Department of Electrical and Electronic Engineering, Khulna University of Engineering & Technology (KUET), Khulna 9203, Bangladesh; selim@eee.kuet.ac.bd

**Keywords:** elliptic curve cryptography (ECC), elliptic curve point multiplication (ECPM), twisted Edwards curve, unified point addition, simple power analysis (SPA) attacks

## Abstract

With the swift evolution of wireless technologies, the demand for the Internet of Things (IoT) security is rising immensely. Elliptic curve cryptography (ECC) provides an attractive solution to fulfill this demand. In recent years, Edwards curves have gained widespread acceptance in digital signatures and ECC due to their faster group operations and higher resistance against side-channel attacks (SCAs) than that of the Weierstrass form of elliptic curves. In this paper, we propose a high-speed, low-area, simple power analysis (SPA)-resistant field-programmable gate array (FPGA) implementation of ECC processor with unified point addition on a twisted Edwards curve, namely Edwards25519. Efficient hardware architectures for modular multiplication, modular inversion, unified point addition, and elliptic curve point multiplication (ECPM) are proposed. To reduce the computational complexity of ECPM, the ECPM scheme is designed in projective coordinates instead of affine coordinates. The proposed ECC processor performs 256-bit point multiplication over a prime field in 198,715 clock cycles and takes 1.9 ms with a throughput of 134.5 kbps, occupying only 6543 slices on Xilinx Virtex-7 FPGA platform. It supports high-speed public-key generation using fewer hardware resources without compromising the security level, which is a challenging requirement for IoT security.

## 1. Introduction

The Internet of Things (IoT) refers a global network, where billions of devices are connected through the Internet and share data with each other. Since most of these devices have constrained resources, data are usually stored in the cloud, where people can continuously upload and download data from anywhere via the Internet [1]. Security concerns arise as data owners have no control over the data management in the cloud-computing environment. The importance of data security and the limited resources of IoT devices motivate us to install lightweight cryptographic schemes that can satisfy the security, low-energy, and low-memory requirements of the existing IoT applications.

Elliptic curve cryptography (ECC), a public-key cryptography (PKC), has become a promising approach to the IoT security, smart card security, and digital signatures as it provides high levels of security with smaller key sizes. Compared with traditional Rivest–Shamir–Adleman (RSA) algorithm, ECC provides an equal level of security but with a shorter key length [2,3,4]. ECC can be implemented with low hardware resource usage and low energy consumption without degrading its security level. Owing to low hardware use, it is well suited for the security of low-power, low-memory, and resource-constrained IoT devices. ECC implemented in a small chip can provide high-speed data encryption and decryption facilities. In addition, it prevents unauthorized devices from gaining access to wireless sensor networks (WSNs) by providing a key agreement protocol for the wireless sensor nodes connected to the IoT infrastructures in the networks [5,6,7,8]. An elliptic curve cryptosystem would be one of the best candidates to meet the privacy and security challenges emerged in radio-frequency identification (RFID) technologies [9,10,11]. Presently, ECC-based untraceable RFID authentication protocols are used in smart healthcare environments to enhance medical data security [12,13,14]. Elliptic curve-based digital signature schemes such as elliptic curve digital signature algorithm (ECDSA) [2] and Edwards curve digital signature algorithm (EdDSA) [15,16] are adopted in wireless body area networks (WBANs) to fulfill the security requirements for real-time health data (e.g., blood pressure, heart rate, and pulse) management [17,18,19]. Modern security protocols such as transport layer security (TLS) and datagram transport layer security (DTLS) deploy these signature schemes for the energy efficient mutual authentication of the servers and clients in IoT platforms [20,21,22].

**Figure 1:**
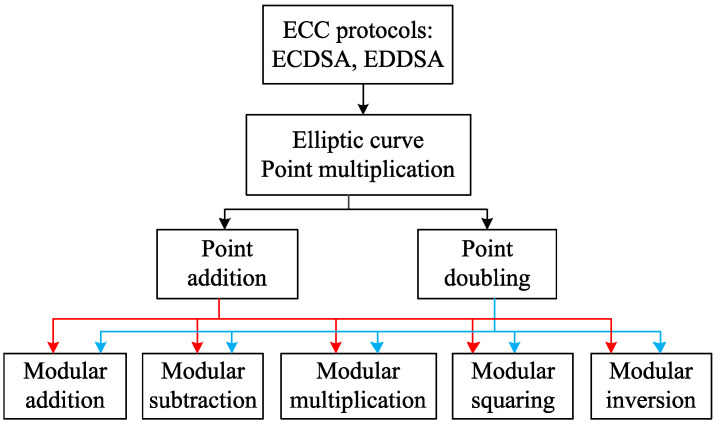
Hierarchy of elliptic curve cryptography.

An ECC hierarchy is equipped with four consecutive levels as shown in Figure 1. The first level contains finite field arithmetic, such as addition, subtraction, multiplication, squaring, and inversion, which can be performed in both the Galois binary field GF($2^{n}$) and Galois prime field GF(*p*). The second level incorporates elliptic curve group operations, such as point addition (PA) and point doubling (PD). In the third level, elliptic curve point multiplication (ECPM) is accomplished by combining the elliptic curve group operations in a sequential manner. The top level includes ECC protocols such as ECDSA and EdDSA. The central and the most time-consuming operation in an elliptic curve-based cryptographic system is ECPM. The principle of ECPM can be specified as Q=kP, where *P* is a base point on an elliptic curve, *k* is a nonzero positive integer, and *Q* is another point on the curve [23]. *Q* and *k* are considered to be public key and private key, respectively, and *P* is regarded as the public-key generator. The retrieval of *k* knowing the points *P* and *Q* is known as elliptic curve discrete logarithm problem (ECDLP) [2] that measures the security strength of the ECPM operation and finds out the weaknesses of the system. The easiest technique to accomplish ECPM is the binary/double-and-add (DAA) algorithm [2] that requires fewer hardware resources compared with other available methods. Therefore, ECC schemes adopting the DAA-based ECPM are suited for IoT applications because of their lower hardware resource requirements and lower power consumption. The major disadvantage of the DAA method is that the DAA-based ECPM is vulnerable to simple power analysis (SPA) attacks [24,25] unless it uses unified point operations.

Edwards curves, a family of elliptic curves, are gaining enormous attention among security researchers because of their simplicity and high resistance against SCAs [26]. ECPM on Edwards curves is faster and more secure than that on the Weierstrass form of elliptic curves [27,28]. Edwards curves have the advantage of providing strongly unified addition formulas [28], which cover both PA and PD. Separate hardware architectures for PA and PD are not required to perform ECPM. Moreover, unified PA prevents probable SPA attacks by making the secret key indistinguishable from power tracing. When ECPM adopts the same module for PA and PD, the binary bit pattern of the secret key cannot be retrieved by SPA. The twisted Edwards curves are a generalization of Edwards curves [29], which are mainly used in the digital signature scheme EdDSA. One of the most compatible twisted Edwards curves in digital signature systems is Edwards25519, which is the Edwards form of the elliptic curve Curve25519 [23,30]. In modern times, Edwards25519 curve is used for a high-speed, high-security digital signature scheme called Ed25519 [15,16]. ECPM using unified twisted Edwards curve not only provides high resistance against SPA but also it reduces the area of ECC processors.

ECC can be accomplished with both hardware and software approaches. Although the software implementation is simple and cost-effective, it cannot provide high-speed computation as the hardware implementation can. Indeed, the hardware implementation of ECC with limited resources is a highly challenging task because low hardware use leads to a lower computational speed. In this point of view, Edwards curves are more effective than classical elliptic curves as they can be implemented on a smaller area with higher processing speed. Most of the hardware implementations of ECC reported in the literature are based on the Weierstrass form of elliptic curves. Few hardware implementations based on twisted Edwards curves over GF(*p*) have been reported. Baldwin et al. [31] first documented hardware implementation of a reconfigurable 192-bit ECC processor adopting twisted Edwards curve over GF(*p*). They provide a comparison between the FPGA implementation of an elliptic curve-based point multiplication and that of a twisted Edwards curve for different number of arithmetic logic units (ALUs) operated in parallel, which shows the Edwards curve as more efficient. Additionally, the twisted Edwards curve point operations are compared with the unified version of these operations. Although the unified version shows little bit worse performance, it provides a higher resistance against SPA. Liu et al. [21] present a computable endomorphism on twisted Edwards curves to boost the speed of ECDSA verification process. They provide area-efficient hardware architecture for signature verification with its FPGA implementation. Application specific integrated circuit (ASIC) implementation of the architecture is also provided for low-cost applications. The implementation results show that the design reduces approximately 50% of the number of PD operations required. Parallel architectures for ECPM on extended twisted Edwards are proposed by Abdulrahman et al. [32]. The authors present a new radix-8 ECPM algorithm to cope with SCAs and speed up computations. However, no hardware implementation of these architectures is reported.

In this paper, a lightweight FPGA-based hardware implementation of ECC over GF(*p*) is proposed for IoT appliances. The major contributions of this paper are summarized as follows:

- An efficient radix-4 interleaved modular multiplier is proposed to perform 256-bit modular multiplication over a prime field.
- A novel hardware architecture for strongly unified PA on the Edwards25519 curve is proposed.
- An efficient ECPM scheme is proposed to perform high-speed point multiplication on the Edwards25519 curve. The same module is used for PA and PD to prevent probable SPA attacks. The area required by the scheme is significantly lower than other available designs for ECPM.
- ECPM is performed in projective coordinates to avoid the most expensive (in terms of computational complexity) modular division operation. In addition, a projective-to-affine (P2A) converter is proposed to transform the projective output into its affine form. This type of transformation reduces the computation time additionally required for the modular division operation performed in affine coordinate-based PA.
- An ECC processor is designed by combining the ECPM scheme and the P2A converter in such a manner as to reduce the number of modular inversion operations required. The area-delay product of the proposed ECC processor is considerably small that ensures a better performance of our processor.

The rest of this paper is organized as follows:

Section 2 presents the mathematical background of the twisted Edwards curve and unified PA formula. Section 3 presents the proposed hardware architectures for field operations (modular multiplication and modular inversion), unified PA, ECPM, and ECC processor. Section 4 presents the implementation results of the proposed designs. Section 5 shows a performance comparison of our proposed ECC processor with other related processors. Finally, Section 6 concludes this research study.

## 2. Mathematical Background

This section presents the twisted Edwards curve with its affine and projective representations as well as the unified PA formula for the curve.

### 2.1. Twisted Edwards Curve

The affine representation of a twisted Edwards curve over a prime field $F_{p}$ with not characteristic 2 is given by the equation [23,29]:

(1) $$t_{a,d}:ax^{2}+y^{2}=1+dx^{2}y^{2},$$

where $a,b\in F_{p}\backslash \{0,1\}$ with a≠d. When a=1, the curve is called untwisted Edwards curve or, formally, Edwards curve. In the case of a=−1, the curve will be

(2) $$t_{d}:-x^{2}+y^{2}=1+dx^{2}y^{2}.$$

when a=−1, d=−121665/121666, and $p=2^{255}-19$, the curve is called Edwards25519 that is the Edwards form of the elliptic curve Curve25519 [23].

In a projective or Jacobian coordinate system, each point (x,y) on $t_{a,d}$ is represented by a triplet form (X,Y,Z). The affine point P(x,y) corresponds to the projective point P(X=x,Y=y,Z=1). The projective point P(X,Y,Z) corresponds to the affine point P(x=X/Z,y=Y/Z) with Z≠0.

The projective representation of the curve $t_{a,d}$ is given by the equation [23,29]:

(3) $$T_{a,d}:\left(aX^{2}+Y^{2}\right)Z^{2}=Z^{4}+dX^{2}Y^{2}.$$

The projective form of the curve $t_{d}$ is given by the equation:

(4) $$T_{d}:\left(-X^{2}+Y^{2}\right)Z^{2}=Z^{4}+dX^{2}Y^{2}.$$

### 2.2. Unified Point-Addition Formula

PA on the curve $T_{d}$ in projective coordinates is given by the equation:

(5) $$P_{1}\left(X_{1},Y_{1},Z_{1}\right)+P_{2}\left(X_{2},Y_{2},Z_{2}\right)=P_{3}\left(X_{3},Y_{3},Z_{3}\right).$$

where $P_{1}$ and $P_{2}$ are two points on the curve and $P_{3}$ is the resultant point.

The unified PA formula [29] for $T_{d}$ can be given as follows:

(6) $$\begin{array}{cc} \; & A=X_{1}X_{2},B=Y_{1}Y_{2},C=Z_{1}Z_{2},D=X_{1}Y_{2},\\ \; & E=X_{2}Y_{1},F=AB,G=A+B,\\ \; & H=C^{2},I=D+E,J=dF,K=CG,\\ \; & L=IC,M=H+J,N=H-J,\\ \; & X_{3}=LN,Y_{3}=MK,Z_{3}=MN. \end{array}$$

The above formula is applicable for both PA and PD. PD can be performed considering that the points $P_{1}$ and $P_{2}$ are identical.

## 3. Proposed Hardware Architectures

This Section presents the proposed hardware architectures for ECC operations and the final ECC processor.

### 3.1. Modular Multiplication

**Figure 2:**
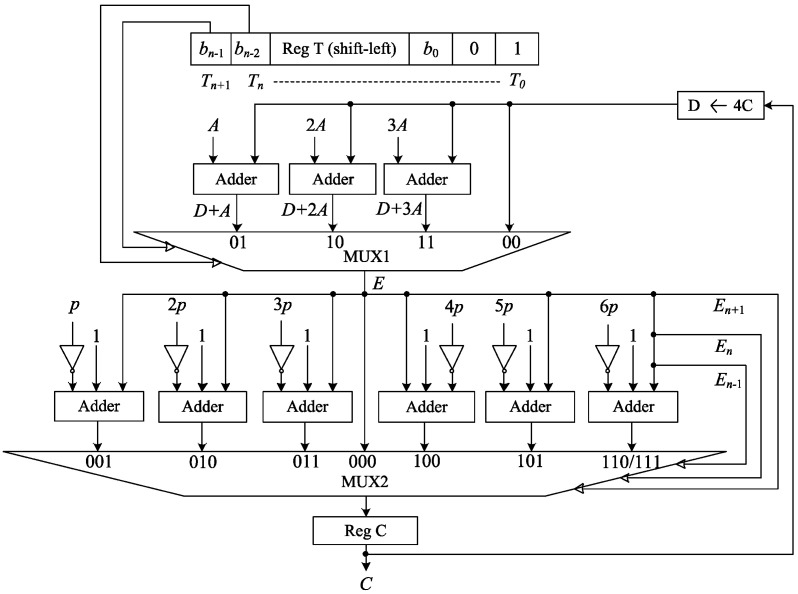
Proposed modular multiplier.

Modular multiplication is the most important arithmetic operation of an ECC processor. The speed and occupied area of the processor entirely depend on it. Although a radix-2 multiplier consumes less hardware resources compared to higher radix (e.g., radix-4 and radix-8) multipliers [33], it is not compatible for high-speed multiplication because of its high latency. To reduce the latency, an efficient radix-4 interleaved modular multiplication algorithm is proposed as demonstrated in Algorithm 1. It requires n/2+1 clock cycles (CCs) to multiply two *n*-bit integers *A* and *B* over the prime field GF(*p*), where *p* is an *n*-bit prime number. Figure 2 illustrates the proposed modular multiplier based on this algorithm.

**Algorithm 1** Proposed Radix-4 Interleaved Modular Multiplication
$\mathrm{Input}:\;A=\sum_{i=0}^{n-1}a_{i}2^{i},B=\sum_{i=0}^{n-1}b_{i}2^{i};a_{i},b_{i}\in \left\{0,1\right\}$

Output:C=(A·B)modp
1:C←0;2:T←B||"01";3:
**while** T(n−1downto0)≠0 **do**
4:   D←4C;5:   **if** T(n+1downton)="01" **then**6:      E←D+A;7:   **else if** T(n+1downton)="10" **then**8:      E←D+2A;9:   **else if** T(n+1downton)="11" **then**10:      E←D+3A;11:   **else**12:      E←D;13:   **end if**;14:   C←Emodp;15:   T←T(n−1downto0)||"00"; \\left shift operation16:**end while**;17:**return***C*;


Modular multiplication is obtained by performing iterative addition of its interim partial products reducing to modulo *p*. A shift-left register “Reg T” is used to perform left to right bitwise multiplication and for a synthesizable loop operation. T[(n+1):2] is precomputed as the multiplier *B* and T[1:0] is precomputed as “01”. These two extra bits are added at the rightmost position of the register T to determine the appropriate end of the loop in the case of $b_{0}=0$. At the beginning of each iteration, accumulator *C* is quadrupled and computed as *D*. For the bitwise multiplication, *A*, 2A, and 3A are separately added to *D*. MUX1 is used to select one of the four outputs D,D+A,D+2A, and  D+3A as *E* based on the three bits T[(n+1):n]. If $T_{n+1}$ and $T_{n}$ both are zero, *D* remains unchanged and *E* becomes *D*. At the end of each iteration, *E* is reduced to modulo *p* and *T* is shifted to the left by 2 bits. The modulo operation (Emodp) is performed by subtracting the prime numbers *p* to (j−1)p from *E*, where *E* is always less than jp;(j=3,4,5...). In this module, (Emodp) is obtained by subtracting the prime numbers *p* to 6p from *E* as *E* is always less than 7p. These subtractions are executed using the 2’s complement method. MUX2 selects one of the seven outputs E,E−p,E−2p,E−3p,E−4p,E−5p, and E−6p as *C* for the next iteration based on the comparisons E≥p,E≥2p,E≥3p,E≥4p,E≥5p, and E≥6p. These comparisons are obtained by checking the three bits E[(n+1):(n−1)]. After n/2 number of iterations, *B*, as well as T[(n−1):0], is shifted to zero value and the execution is stopped. The final content of the register “Reg C” is the modular multiplication of *A* and *B*.

A total of n/2+1 CCs are required to perform the modular multiplication operation, where n/2 CCs correspond to n/2 number of iterations and one extra CC is required for the initialization. To perform modular squaring, the inputs *A* and *B* are taken as identical.

### 3.2. Modular Inversion

**Figure 3:**
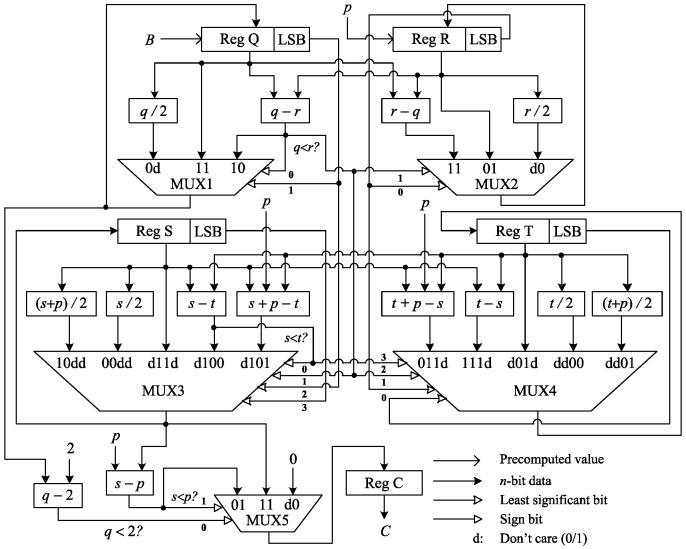
Proposed hardware architecture for modular inversion.

Modular inversion is the costliest (in terms of the hardware resource requirements) arithmetic operation in finite fields. In affine representations, PA and PD require modular inversion operation to perform modular division. In this study, although our ECC processor is designed in projective coordinates, modular inversion is required for P2A conversion. Algorithm 2 [2] demonstrates the binary modular inversion for the P2A conversion module proposed in this paper. The hardware architecture of this module is depicted in Figure 3.
**Algorithm 2** Binary Modular Inversion [2]$\mathrm{Input}:\;B=\sum_{i=0}^{n-1}b_{i}2^{i};b_{i}\in \left\{0,1\right\}$ $\mathrm{Output}:\;C=B^{-1}\;mod\;p$1:C←0,q←B,r←p,s←1,t←0;2:**while**q≠1**do**3:   **while** q(0)=0 **do**4:       q←q/2;5:       **if** s(0)=0 **then**6:          s←s/2;7:       **else**8:          s←(s+p)/2;9:       **end if**;10:   **end while**;11:   **while** r(0)=0 **do**12:       r←r/2;13:       **if** t(0)=0 **then**14:          t←t/2;15:       **else**16:          t←(t+p)/2;17:       **end if**;18:   **end while**;19:   **if** q>r **then**20:       q←q−r;21:       **if** s>t **then**22:          s←s−t;23:       **else**24:          s←s+p−t;25:       **end if**;26:   **else**27:       r←r−q;28:       **if** t>s **then**29:          t←t−s;30:       **else**31:          t←t+p−s;32:       **end if**;33:   **end if**;34:**end while**;35:**return**smodp;

The contents of the registers “Reg Q”, “Reg R”, “Reg S”, and “Reg T” are updated in every iteration. Five multiplexers such as MUX1, MUX2, MUX3, MUX4, and MUX5 are used to select corresponding outputs, satisfying different conditions by their select lines. In the case of *q* being even, MUX1 selects q/2 and MUX3 selects s/2 if *s* is even or (s+p)/2 if *s* is odd. In the case of *q* being odd and greater than *r*, MUX1 selects q−r and MUX3 selects s−t if s>t or s+p−t if s<t. The comparisons q>r and s>t are obtained by checking the sign bits of the subtractions q−r and s−t, respectively. If *q* is odd and less than *r*, *q* and *r* both remain unchanged. Similarly, MUX2 selects one of the three outputs *r*, r/2, and r−q based on the conditions r(0)=0 and r>q. MUX4 selects one of the five outputs *t*, t/2, (t+p)/2, t−s, and t+p−s based on the conditions r(0)=0, t(0)=0, r>q, and t>s. MUX5 is used to select the final result as (smodp) if q=1. In this regard, *q* is subtracted by 2 to check whether q<2 at the end of each iteration. When the sign bit of the subtraction q−2 is 1, (smodp) is stored in the register “Reg C”, which is the modular inversion of *B*.

In this architecture, on average n+n/4 CCs are required to perform the modular inversion operation, where *n* number of iterations are to reduce the *n*-bit variable *q* to 1 in a regular manner and additional n/4 number of iterations are for such uncertain case as *q* being odd. The clock cycles required for the modular inversion operation may vary from our estimation depending on the binary bit pattern of *B*.

### 3.3. Unified Point Addition

**Figure 4:**
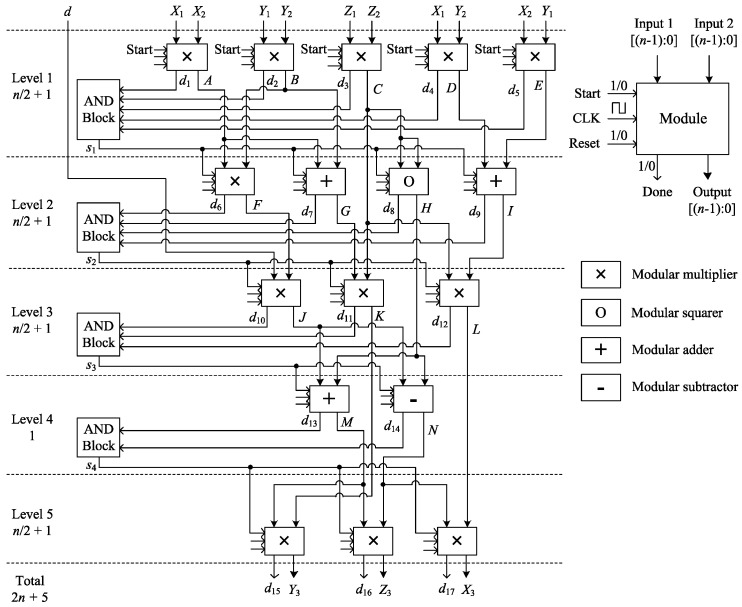
Proposed hardware architecture for unified PA.

Unified PA is required to perform both PA and PD by the same module so as to prevent possible SPA attacks in ECPM. The proposed hardware architecture for the unified PA formula described in (6) is depicted in Figure 4. The architecture includes 12 multiplications, 1 squaring, 3 additions, and 1 subtraction, which can be denoted as (12M+1S+4A). The proposed design consists of four consecutive levels, in which the arithmetic modules are connected in a sequential manner. The modules are arranged in horizontally parallel among the levels to achieve the shortest data path. The whole architecture is efficiently balanced to reduce the area required. Start signals are used to start the arithmetic operations and Done signals are used to confirm the end of the operations. The Done signals of the modules at each level are considered to be the Start signals of the modules at its subsequent level. AND blocks are used to synchronize the horizontal modules in time (e.g., if the Done signals $d_{1},d_{2},d_{3},d_{4}$, and $d_{5}$ all be 1, the Start signal $s_{1}$ will be 1; otherwise, it will be 0). The modular multiplier and the squarer require n/2+1 CCs to perform modular multiplication and squaring. Modular addition and subtraction are completed in only one CC. The level that contains any multiplication or squaring operation requires n/2+1 CCs and the level that contains no multiplication or squaring requires one CC to jump to the next level. In this design, a total of 2n+5 CCs are required to complete the unified PA operation.

### 3.4. Elliptic Curve Point Multiplication

ECPM is the ultimate operation of an ECC processor. It multiplies a point on an elliptic curve with a scalar. The execution time of ECC schemes is dominated by ECPM. Let P(X,Y,Z) be a point on the curve $T_{d}$, *k* be a scalar that is considered to be secret key. A  public key Q(X,Y,Z) is generated from the known base point *P* and the secret key *k* by performing ECPM as follows:

(7) Q=kP,

where *Q* is also a point on the curve. It can be obtained by adding *P* to itself k−1 times such as

(8) $$Q=P+\underset{k-1\;\mathrm{times}}{\underset{︸}{P+.......+P.}}$$

If *k* is expressible as a power of 2, *Q* can be obtained by doubling *P* on itself $log_{2}k$ times such as

(9) $$Q=\underset{log_{2}k\;\mathrm{times}}{\underset{︸}{...2(2(2(P))).}}$$

**Figure 5:**
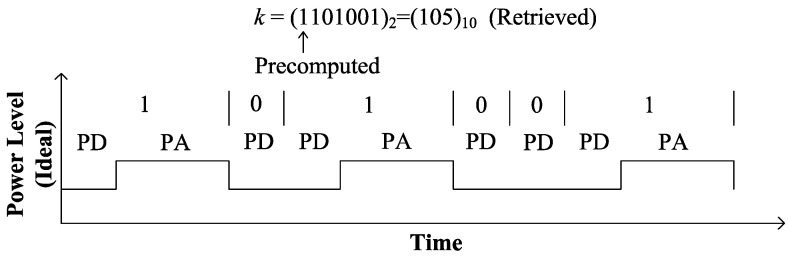
Simplistic representation of SPA in conventional DAA ECPM.

**Figure 6:**
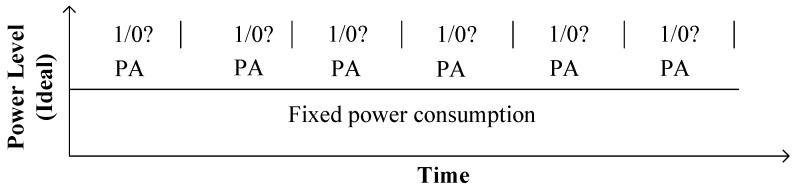
Simplistic representation of SPA in proposed unified PA-based ECPM.

**Figure 7:**
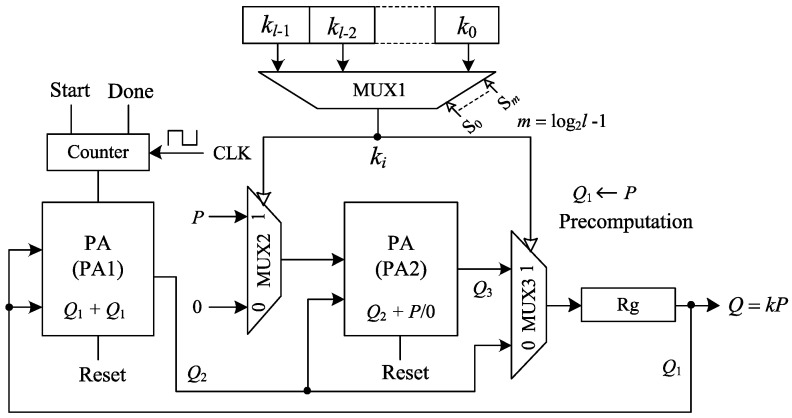
Proposed hardware architecture for ECPM.

In the binary/ DAA method, ECPM is performed by a combination of PD and PA following the binary bit pattern of the secret key as shown in Algorithm 3. In this algorithm, separate modules are required to perform PA and PD. The power consumption of the two separate modules are different. Monitoring these two power levels by SPA, the bit pattern of *k* can be retrieved as shown in Figure 5. Moreover, *k* can be assumed by timing analysis; hence, ECPM based on this algorithm is vulnerable to SPA attacks. To cope with SPA, Algorithm 3 is modified to Algorithm 4, where PD is replaced by unified PA. According to this algorithm, power is only consumed for PA with a fixed power consumption, which is independent of the bit pattern of *k* as shown in Figure 6. Since the power consumption is the same across all the iterations, this algorithm is free from SPA. Figure 7 illustrates the proposed hardware architecture for ECPM based on Algorithm 4. Two point-addition blocks PA1, PA2 and three multiplexers MUX1, MUX2, MUX3 are used in this processor. Initially, $Q_{1}$ is precomputed as *P*. PA1 adds the point $Q_{1}$ to itself and the output $Q_{2}$ goes to the input of PA2. Identical inputs are inserted in PA1 to perform PD by means of PA. One of the two inputs of PA2 is the output of PA1 and the other one is *P* or 0. If $k_{i}=1$, PA2 adds the point *P* to the point $Q_{2}$ and the output $Q_{3}$ goes to the input of the PA1 via the register Rg. On the contrary, if $k_{i}=0$, PA2 remains idle and the output of PA1 directly goes to its input via Rg. MUX1 is used to select the *i*th bit of *k* by $log_{2}l$ number of select lines, where *l* is the bit length of *k*. Based on $k_{i}$, MUX2 selects *P* or 0 as one of the two inputs of PA2; MUX3 selects $Q_{2}$ or $Q_{3}$ as the input $Q_{1}$ for the subsequent iteration.

**Algorithm 3** DAA ECPM without Unified PA [2]
$\mathrm{Input}:\;P\left(X,Y,Z\right),k=\sum_{i=0}^{l-1}k_{i}2^{i};k_{i}\in \left\{0,1\right\},k_{l-1}=1$

Output:Q(X,Y,Z)
1:Q←P;2:
**for** ifroml−2to0 **do**
3:   Q←2Q;          \\PD4:   **if** $k_{i}=1$ **then**5:       Q←Q+P;    \\PA6:   **end if**;7:**end for**; 8:**return***Q*;


**Algorithm 4** Proposed Unified PA-based ECPM
$\mathrm{Input}:\;P\left(X,Y,Z\right),k=\sum_{i=0}^{l-1}k_{i}2^{i};k_{i}\in \left\{0,1\right\},k_{l-1}=1$

Output:Q(X,Y,Z)
1:$Q_{1}\leftarrow P$;2:
**for** ifroml−2to0 **do**
3:     $Q_{2}\leftarrow Q_{1}+Q_{1}$;     \\PA4:     **if** $k_{i}=1$ **then**5:         $Q_{3}\leftarrow Q_{2}+P$;    \\PA6:         $Q_{1}\leftarrow Q_{3}$;7:     **else**8:         $Q_{1}\leftarrow Q_{2}$;9:     **end if**10:
**end for**
11:**return**$Q_{1}$;


For the *l*-bit *k*, the register stores kP as the final result after l−1 number of iterations. The average CCs required to perform the ECPM can be calculated as

(10) $$\begin{array}{cc} {\mathrm{ECPM}}_{\mathrm{CC}} & =\left(l-1\right)\times \left({\mathrm{PA}1}_{\mathrm{CC}}+{\mathrm{Rg}}_{\mathrm{CC}}\right)+l/2\times {\mathrm{PA}2}_{\mathrm{CC}}\\ \; & =(l-1)\times (2n+5+1)+(l/2)\times (2n+5)\\ \; & =3nl-2n+8.5l-6. \end{array}$$

For l=n,

(11) $$\begin{array}{c} {\mathrm{ECPM}}_{\mathrm{CC}}=3n^{2}+6.5n-6. \end{array}$$

PA1 and Rg remain active in every iteration, whereas PA2 goes idle in the case of $k_{i}=0$. In every iteration, a total 2n+6 CCs are spent by PA1 and Rg. Additional 2n+5 CCs are spent by PA2 if $k_{i}=1$. On average, l(n+2.5) CCs are spent by PA2 across the ECPM. For the *n*-bit *k*, the latency of the ECPM is approximately $3n^{2}+6.5n-6$ CCs. This latency may vary depending on the bit pattern of the key; it increases with the number of 1 and decreases with the number 0 present in the bit pattern. In this study, an average case is considered. This means that the key has equal number of 1 and 0 in its bit pattern, although this is not always the case.

### 3.5. Proposed ECC Processor

**Figure 8:**
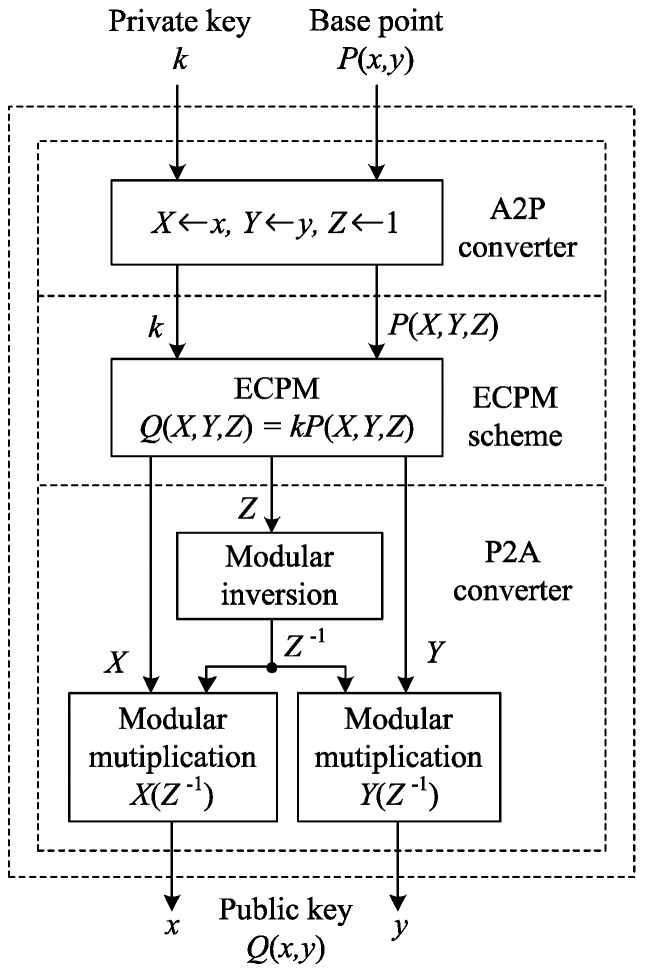
Proposed ECC processor for public-key generation.

A time-area-efficient ECC processor is designed for public-key generation using the proposed projective coordinate-based ECPM along with a P2A converter as shown in Figure 8. This processor will generate a public key from a private key and a base point on $T_{d}$. Initially, the affine base point P(x,y) is transformed into its projective form such as P(X,Y,Z) by an affine-to-projective (A2P) converter. The public key Q(X,Y,Z) is obtained by performing ECPM of the projective point P(X,Y,Z) with the secret key *k*. Finally, Q(X,Y,Z) is transformed into its affine form such as Q(x,y) by the P2A converter. For the P2A conversion, *Z* is inverted by the proposed modular inversion module and separately multiplied by *X* and *Y*. The latency required by the processor to process the ECPM operation along with the coordinate conversions is $3n^{2}+8.25n-5$ CCs, which is the total sum of the latency of ECPM, modular inversion, and modular multiplication.

## 4. Implementation Results

The proposed ECC processor was programmed in VHDL and implemented using the Xilinx ISE 14.7 Design Suite software. Xilinx ISim simulator was used to simulate the ECC operations. The simulation results were verified by the Maple 18 software. Synthesizing, mapping, placing, and routing of the proposed ECC modules were performed on Xilinx Virtex-7 and Virtex-6 FPGA platforms, separately. The details of these FPGA platforms and settings are as follows:

- Platform 1: Virtex-7 (XC7VX690T)
- Platform 2: Virtex-6 (XC6VHX380T)
- Design Goal: Balanced
- Design Strategy: Xilinx Default
- Optimization Goal: Speed
- Optimization Effort: Normal

The implementation results of the proposed ECC modules are summarized in Table 1. On Platform 1, all the modules run at a maximum frequency of 104.39 MHz. The proposed ECC processor occupies 6543 slices (25,898 LUTs) and generates a public key from a given 256-bit private key in 1.9 ms with a throughput of 134.5 kbps. On Platform 2, the modules operate at a maximum frequency of 93.23 MHz. The numbers of slices and LUTs used by the processor are 6579 and 25,968, respectively, the delay of the public-key generation is 2.13 ms, and the throughput is 120.1 kbps.

The performance of the ECC modules on the Virtex-6 FPGA platform is a little bit worse compared to the Virtex-7 FPGA platform in terms of speed. However, the area use of the different modules on these platforms are almost the same. It must be noted that no digital signal processing (DSP) slice is used to implement our processor. Although DSP slices increase processing speed, they increase processor’s cost as well.

## 5. Performance Comparison

Several hardware implementations of ECC have been reported in [34,35,36,37,38,39,40,41,42,43,44,45,46,47,48,49,50,51,52,53], where some authors aimed to minimize the area use while others tried to reduce the computation time. Achieving a higher processing speed with low-area use is technically challenging. We tried to maintain a balance between area and time as they are two important performance criteria of a cryptographic processor. A performance comparison of our proposed ECC processor with other related designs is presented in Table 2.

The residue number system (RNS)-based ECC design reported in [34] provides a higher throughput (1816.2 kbps) by performing ECPM on 21 keys in parallel. Conventional DAA method is adopted for ECPM, where PA and PD are executed by separate modules carrying high risk of SPA attacks. On Virtex-7 FPGA, the design consumes 96,867 LUTs (approx. 24,216 slices) with 2799 additional DSP slices. Although the throughput of this design is higher than that of our design, it costs 3.7 times more hardware resources. The novelty of this design is that it processes 21 keys simultaneously, which prevents template-based attacks by increasing the computation complexity. In [35], the authors propose a high-performance ECC processor with its ASIC and FPGA implementations. A novel hardware architecture for combined PA-PD operation in Jacobian coordinates is proposed to achieve high-speed ECPM with low hardware use. On Kintex-7 FPGA, the processor separately designed in affine and Jacobian coordinates performs ECPM in 4.7 ms and 3.27 ms, occupying 9.3k and 11.3k slices, respectively. Our processor implemented on 7-series FPGA is 1.72 times faster and costs 1.73 times less slices as compared with this processor designed in Jacobian coordinates. The throughput of our design is 1.76 times higher. A high-speed ECC processor is proposed in [36] providing redundant signed digit (RSD)-based carry free modular arithmetic. The processor performs high-speed ECPM with a higher throughput. However, it occupies 10 times more slices on Virtex-6 FPGA than our processor. Although RSD representation offers fast computation, it consumes a vast amount of hardware resources, which makes processor bulky and hence not suited for low-power IoT devices. The high-speed RSD-based modular multiplier proposed in this paper performs single multiplication in only 0.34 μs, consuming 22k LUTs. In comparison with this multiplier, our proposed modular multiplier performs single multiplication in 1.45 μs and consumes only 1.3k LUTs with almost 4 times better efficiency in terms of area-time (AT) product. The RSD-based ECC processors reported in [37,38] present comprehensive pipelining technique for Karatsuba–Ofman multiplication to achieve high throughput. Our processor has smaller AT product compared with these processors.

Liu et al. [39] propose a hardware-software approach for flexible duel-field ECC processor with its ASIC and FPGA implementations. The traditional DAA method for ECPM is replaced by the double-and-add-always (DAAA) method to protect the processor from SPA attacks. Although the DAAA method for ECPM provides high resistance against SPA, it increases the computational complexity and hence reduces the frequency and throughput. In addition, it consumes more power than the conventional DAA method as PA and PD are performed in every iteration. Our processor is protected against SPA attacks by implementing the cost-effective DAA algorithm with unified PA. When compared to our processor, the main advantage of this processor is that it is flexible and reconfigurable over different field orders. In addition, it can perform ECPM over both GF($2^{n}$) and GF(*p*), whereas our processor performs ECPM over GF(*p*) only.

Hu et al. [40] propose an SPA-resistant ECC design over GF(*p*), providing its ASIC and FPGA implementations. The design uses 9370 slices with 14 additional DSP slices on Virtex-4 FPGA. Despite employing additional DSP slices, the speed of this design is considerably low. It takes 29.84 ms with a frequency of 20.44 MHz to perform single ECPM over a 256-bit prime field. The advantage of this design that makes it well suited for embedded applications is its reconfigurable computing capability. A low latency ECPM design is proposed in [41] exploiting parallel multiplication over GF(*p*). Protection against timing and SPA attacks is provided by using the DAAA method for ECPM. The latency of this design is $3n^{2}+37n+4n$ CCs, whereas the latency of our design is $3n^{2}+8.25n-5$ CCs. Therefore, the computational complexity of ECPM in this design is higher than that in our design. The radix-4 parallel interleaved modular multiplier proposed in this paper performs multiplication in 0.79 μs, consuming 6.3k LUTs. Four multiplier units are operated in parallel to speed up the multiplication process. The main feature of this design is its capability to perform ECPM over GF(*p*) with any arbitrary value of *p* less than or equal to 256 bits in size.

The design reported in [42] exploits the Montgomery ladder algorithm for SPA-resistant ECPM. Although the Montgomery ladder algorithm offers lower latency ECPM and higher resistance against SPA than the general DAA method [23], it deals with around 50% additional PA operations that results in a higher power consumption. Hence, the DAA method is more efficient than the Montgomery ladder technique in terms of energy consumption. The advantage of this design is that it supports any prime number p≤256-bit. In [43], the authors present a high-performance hardware design for ECPM adopting non-adjacent form (NAF) method. Although NAF method has the advantage of reducing the latency of ECPM, the computational complexity and its vulnerability to SCAs are high in this method. Moreover, additional point subtraction operation is required for NAF scalar multiplication. Like the designs reported in [40,41], this design is programmable for any prime p≤256-bit. Parallel crypto design is proposed in [44] using the DAAA method to perform SCA-resistant ECPM over different field orders. The design is represented in affine coordinates, where PA and PD require modular division operations. Modular division is the most time-consuming arithmetic operation in finite fields. Therefore, this design is not convenient for high-speed computation. However, it provides high resistance against timing and SPA attacks by parallel computation of PA and PD.

Ananyi et al. [45] propose a flexible hardware ECC processor that supports five National Institute of Standard and Technology (NIST) recommended prime curves. They provide a comparison between the binary and NAF ECPM over all five NIST prime fields such as $p_{192},p_{224},p_{256},p_{384},$ and $p_{521}$, where the NAF ECPM is found to be more time-efficient. Their processor consumes 20,793 slices (31,946 LUTs) with 32 additional DSP blocks on Virtex-4 FPGA and performs the binary ECPM in 6.9 ms and the NAF ECPM in 6.1 ms over $p_{256}$. The modular inverter designed in this paper operates at a frequency of 58.6 MHz costing 10,921 slices with 32 DSP blocks, whereas our modular inverter implemented on Virtex-7 FPGA runs at 110.65 MHz consuming 1197 slices without any DSP block.

A scalable ECC processor developed by Loi et al. [46] can perform ECPM on five NIST suggested prime curves such as P-192, P-224, P-256, P-384, and P-521 without hardware reconfiguring. On Virtex-4 FPGA, this processor performs ECPM in 5.46 ms, occupying 7020 slices along with 8 additional DSP slices. Despite using DSP slices, the computational speeds of the processors reported in [45,46] are low. The main significance of these processors is that they are flexible over the five NIST prime fields and hence they can be programmed to perform ECPM for variable prime numbers ranging from 192 to 521 bits in size without being architecturally reconfigured. The processors reported in [47,48,49,50,51,52,53], are implemented on some backdated FPGA platforms, which are now obsolete.

**Figure 9:**
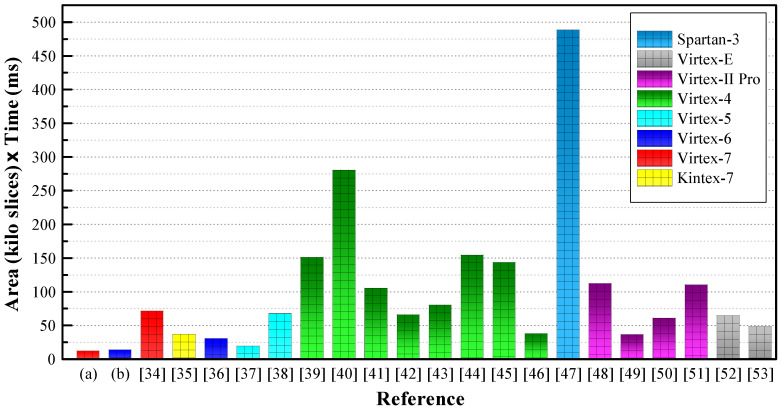
Performance comparison in terms of AT product.

**Figure 10:**
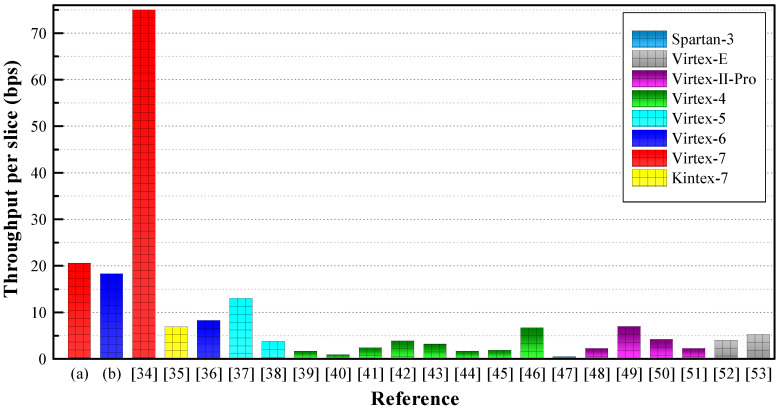
Performance comparison in terms of throughput per slice.

Performance comparison in terms of AT product is shown in Figure 9. The AT product of our design is lower than that of the other designs tabulated in Table 2. Figure 10 shows performance comparison in terms of throughput per slice. The per slice throughput of our design is higher than that of the other designs except [34]. The RNS-based design reported in [34] provides a higher throughput by performing ECPM on 21 keys concurrently. Our processor’s low value of AT product and high value of throughput ensure a better performance in IoT platforms. However, a fair comparison is not possible because the compared processors are implemented on different FPGA platforms. Our proposed ECC processor is implemented only on the Virtex-7 and Virtex-6 FPGAs because the number of input/output blocks (IOBs) is limited in earlier FPGAs. Furthermore, the earlier FPGAs such as Virtex-II-Pro, Virtex-4, and Virtex-5 are not compatible with low-power devices because of their high power consumption.

## 6. Conclusions

In this paper, a high-performance ECC processor has been proposed exploiting unified PA on Edwards25519 curve to perform SPA-resistant point multiplication. An efficient ECPM module has been designed in projective coordinates, which supports 256-bit point multiplication over a prime field. Unified PA is adopted for the ECPM module to provide strong protection against SPA attacks and reduce the area required by an additional PD module. To perform high-speed modular multiplication, an efficient radix-4 interleaved modular multiplier has been proposed. The proposed ECC processor performs fast point multiplication with a considerably lower area use, providing high resistance against SPA. Because of its less hardware resource requirements and high computation speed, it is well suited for resource-constrained IoT devices. Since it provides a faster ECPM that is a rising demand of elliptic curve-based digital signature schemes, it could be manipulated in Bitcoin-like cryptocurrencies for high-speed digital signature generation and verification, which would reduce latency in transaction confirmation. Based on the overall performance analyses, it can be concluded that the proposed ECC processor could be a good choice for the IoT security as well as the emerging technology “Blockchain”.

## Figures and Tables

**Table 1:** Implementation results of the proposed ECC modules on different FPGA platforms over $F_{p}$-256.

Operation	Platform	CCs	Area (Slices)	Area (LUTs)	Maximum Frequency (MHz)	Time (μs)	Throughput (Mbps)
Modular multiplication	Virtex-7	129	416	1451	104.39	1.24	207.2
	Virtex-6	129	420	1460	93.23	1.38	185
Modular inversion	Virtex-7	320	1197	4155	110.65	2.89	83.5
	Virtex-6	320	1209	4156	97.94	3.27	74.6
Unified PA	Virtex-7	517	4159	15,594	104.39	4.95	51.7
	Virtex-6	517	4292	15,593	93.23	5.55	46.2
ECPM (projective)	Virtex-7	198,266	5457	21,194	104.39	1899	$134.8\times 10^{-3}$
	Virtex-6	198,266	5541	21,224	93.23	2126	$120.4\times 10^{-3}$
Public-key generation	Virtex-7	198,715	6543	25,898	104.39	1903	$134.5\times 10^{-3}$
	Virtex-6	198,715	6579	25,968	93.23	2131	$120.1\times 10^{-3}$

**Table 2:** Performance comparison of the proposed ECC processor with other related designs over $F_{p}$-256.

Design	Platform	Area (Slices)	CCs	Frequency (MHz)	Time (ms)	Throughput (kbps)	Area × Time
Ours (a)	Virtex-7	6.5k	198.7	104.39	1.9	134.49 ${}^{a}$	12.35
Ours (b)	Virtex-6	6.6k	198.7	93.23	2.13	120.12 ${}^{a}$	14.05
[34]	Virtex-7	24.2k, 2.8k DSPs	215.9	72.9	2.96	1816.2	71.63
[35]	Kintex-7	11.3k	397.3	121.5	3.27	78.28	36.95
[36]	Virtex-6	65.6k	153.2	327	0.47	546.42 ${}^{a}$	30.83
[37]	Virtex-5	8.7k	361.6	160	2.26	113.27 ${}^{a}$	19.66
[38]	Virtex-5	10.2k	442.2	66.7	6.63	38.61 ${}^{a}$	67.63
[39]	Virtex-4	12k	459.9	36.5	12.6	20.32 ${}^{a}$	151.20
[40]	Virtex-4	9.4k, 14 DSPs	610	20.44	29.84	8.58 ${}^{a}$	280.50
[41]	Virtex-4	35.7k	207.1	70	2.96	86.53 ${}^{a}$	105.67
[42]	Virtex-4	13.2k	200	40	5	51	66.00
[43]	Virtex-4	20.6k	191.6	49	3.91	65.47	80.55
[44]	Virtex-4	20.1k	331.1	43	7.7	33.25 ${}^{a}$	154.77
[45]	Virtex-4	20.8k, 32 DSPs	414	60	6.9	37.10 ${}^{a}$	143.52
[46]	Virtex-4	7k, 8 DSPs	993.7	182	5.46	46.88 ${}^{a}$	38.22
[47]	Spartan-3	27.6k	708	40	17.7	14.46 ${}^{a}$	488.52
[48]	Virtex-II Pro	12k	337.7	36	9.38	27.29 ${}^{a}$	112.56
[49]	Virtex-II Pro	8.3k	163.2	37	4.41	58.04 ${}^{a}$	36.60
[50]	Virtex-II Pro	15.8k, 256 DSPs	151.4	39.5	3.86	66.74	60.98
[51]	Virtex-II Pro	41.6k	252.1	94.7	2.66	96.17 ${}^{a}$	110.66
[52]	Virtex-E	16.4k	156.8	39.7	3.95	64.82 ${}^{a}$	64.78
[53]	Virtex-E	14.2k	118.3	34.7	3.41	75.09 ${}^{a}$	48.42

Estimated by the authors of this paper as Throughput = (Maximum frequency ÷ CCs)× 256.
